# Subgenomic RNA identification in SARS-CoV-2 genomic sequencing data

**DOI:** 10.1101/gr.268110.120

**Published:** 2021-04

**Authors:** Matthew D. Parker, Benjamin B. Lindsey, Shay Leary, Silvana Gaudieri, Abha Chopra, Matthew Wyles, Adrienn Angyal, Luke R. Green, Paul Parsons, Rachel M. Tucker, Rebecca Brown, Danielle Groves, Katie Johnson, Laura Carrilero, Joe Heffer, David G. Partridge, Cariad Evans, Mohammad Raza, Alexander J. Keeley, Nikki Smith, Ana Da Silva Filipe, James G. Shepherd, Chris Davis, Sahan Bennett, Vattipally B. Sreenu, Alain Kohl, Elihu Aranday-Cortes, Lily Tong, Jenna Nichols, Emma C. Thomson, The COVID-19 Genomics UK (COG-UK) Consortium, Dennis Wang, Simon Mallal, Thushan I. de Silva

**Affiliations:** 1 Sheffield Bioinformatics Core, The University of Sheffield, Sheffield S10 2HQ, United Kingdom;; 2 Sheffield Institute for Translational Neuroscience, The University of Sheffield, Sheffield S10 2HQ, United Kingdom;; 3 Sheffield Biomedical Research Centre, The University of Sheffield, Sheffield S10 2JF, United Kingdom;; 4 Sheffield Teaching Hospitals NHS Foundation Trust, Department of Virology/Microbiology, Sheffield S10 2JF, United Kingdom;; 5 The Florey Institute for Host-Pathogen Interactions and Department of Infection, Immunity and Cardiovascular Disease, Medical School, University of Sheffield, Sheffield S10 2TN, United Kingdom;; 6 Institute for Immunology and Infectious Diseases, Murdoch University, Murdoch WA 6150, Western Australia, Australia;; 7 Division of Infectious Diseases, Department of Medicine, Vanderbilt University Medical Center, Nashville, Tennessee 37232, USA;; 8 School of Human Sciences, University of Western Australia, Crawley WA 6009, Western Australia, Australia;; 9 Department of Animal and Plant Sciences, The University of Sheffield, Sheffield S10 2TN, United Kingdom;; 10 IT Services, The University of Sheffield, Sheffield S10 2FN, United Kingdom;; 11 Centre for Virus Research, The University of Glasgow, Glasgow G61 1QH, United Kingdom;; 12 https://www.cogconsortium.uk;; 13 Department of Computer Science, The University of Sheffield, Sheffield S1 4DP, United Kingdom

## Abstract

We have developed periscope, a tool for the detection and quantification of subgenomic RNA (sgRNA) in SARS-CoV-2 genomic sequence data. The translation of the SARS-CoV-2 RNA genome for most open reading frames (ORFs) occurs via RNA intermediates termed “subgenomic RNAs.” sgRNAs are produced through discontinuous transcription, which relies on homology between transcription regulatory sequences (TRS-B) upstream of the ORF start codons and that of the TRS-L, which is located in the 5′ UTR. TRS-L is immediately preceded by a leader sequence. This leader sequence is therefore found at the 5′ end of all sgRNA. We applied periscope to 1155 SARS-CoV-2 genomes from Sheffield, United Kingdom, and validated our findings using orthogonal data sets and in vitro cell systems. By using a simple local alignment to detect reads that contain the leader sequence, we were able to identify and quantify reads arising from canonical and noncanonical sgRNA. We were able to detect all canonical sgRNAs at the expected abundances, with the exception of ORF10. A number of recurrent noncanonical sgRNAs are detected. We show that the results are reproducible using technical replicates and determine the optimum number of reads for sgRNA analysis. In VeroE6 *ACE2*+/− cell lines, periscope can detect the changes in the kinetics of sgRNA in orthogonal sequencing data sets. Finally, variants found in genomic RNA are transmitted to sgRNAs with high fidelity in most cases. This tool can be applied to all sequenced COVID-19 samples worldwide to provide comprehensive analysis of SARS-CoV-2 sgRNA.

Understanding variation within subgenomic RNA (sgRNA) synthesis within the human host may have important implications for the study of SARS-CoV-2 biology and evolution. Owing to advances in sequencing technology and collaborative science, more than 100,000 SARS-CoV-2 genomes have been sequenced worldwide to date.

The genome of SARS-CoV-2 comprises a single positive-sense RNA molecule of ∼29 kb in length. Although the 1a and 1b polyproteins are translated directly from this genomic RNA (gRNA), all other proteins are translated from sgRNA intermediates (Stern and Kennedy 1980; Sola et al. 2015). sgRNAs are produced through discontinuous transcription during negative-strand synthesis followed by positive-strand synthesis to form mRNA. The resulting sgRNAs contain a leader sequence derived from the 5′ untranslated region of the genome and a transcription regulating sequence (TRS) 5′ of the open reading frame (ORF). The template switch occurs during sgRNA synthesis owing to a conserved core sequence within the TRS 5′ of each ORF (TRS-B) and the TRS within the leader sequence (TRS-L) (Zúñiga et al. 2004). The conserved core sequence leads to base-pairing between the TRS-L and the nascent RNA molecule transcribed from the TRS-B, resulting in a long-range template switch and incorporation of the 5′ leader sequence (Sola et al. 2015). SARS-CoV-2 produces at least nine canonical sgRNAs containing ORFs for four structural proteins (S, spike; E, envelope; M, membrane; N, nucleocapsid) and several accessory proteins (3a, 3b, 6, 7a, 7b, 8, and 10) (Davidson et al. 2020; Wu et al. 2020; Zhou et al. 2020). In SARS-CoV ORFs, 3b and 7b are considered nested ORFs and not thought to be translated from their own sgRNA (Inberg and Linial 2004). Understanding variation within sgRNA synthesis within the human host may have important implications for the study of SARS-CoV-2 biology and evolution.

Beyond the regulation of transcription, sgRNA may also play a role in the evolution of coronaviruses, and the template switching required for sgRNA synthesis may explain the high rate of recombination seen in coronaviruses (Wu and Brian 2010; Simon-Loriere and Holmes 2011). Whereas the majority of sgRNA relate to known ORFs, novel, noncanonical sgRNA are also produced (Finkel et al. 2020; Kim et al. 2020; Nomburg et al. 2020), although the biological function of this is unclear. sgRNAs have also been shown to modulate host cell translational processes (Patel et al. 2013).

It has previously been shown that sgRNA transcript abundances can be quantified from full RNA-seq data by calculation of reads per kilobase of transcript, per million mapped reads (RPKM) or by using so-called “chimeric” fragments containing the leader and TRS (Irigoyen et al. 2016). From two independent repeats, the *R*^2^ between these two measurement methods was 0.99.

The ARTIC Network (2020) protocol for the sequencing of the SARS-CoV-2 (Fig. 1A) has been used worldwide to characterize the genetic diversity of this novel coronavirus. The COVID-19 Genomics UK (COG-UK) Consortium (2020) in the UK, alone, has produced 16,826 ARTIC Nanopore genome sequences (correct October 29, 2020), whereas internationally GISAID contains thousands more similar data sets (8775 with “Nanopore” in the metadata and 3660 list “artic,” June 14, 2020). This protocol involves the amplification of 98 overlapping regions of the SARS-CoV-2 genome in two pools of 49 amplicons to provide full sequence coverage when sequenced with Oxford Nanopore sequencing devices. All known SARS-CoV-2 ORF TRS sites are contained within one or more amplicons in this panel (Fig. 1A). Other methods of enrichment and enrichment-free sequencing of the SARS-CoV-2 genome like bait-based capture and subsequent short-read Illumina sequencing or metagenomics, respectively, are also popular and hold promise for the detection of sgRNA.

**Figure 1.**
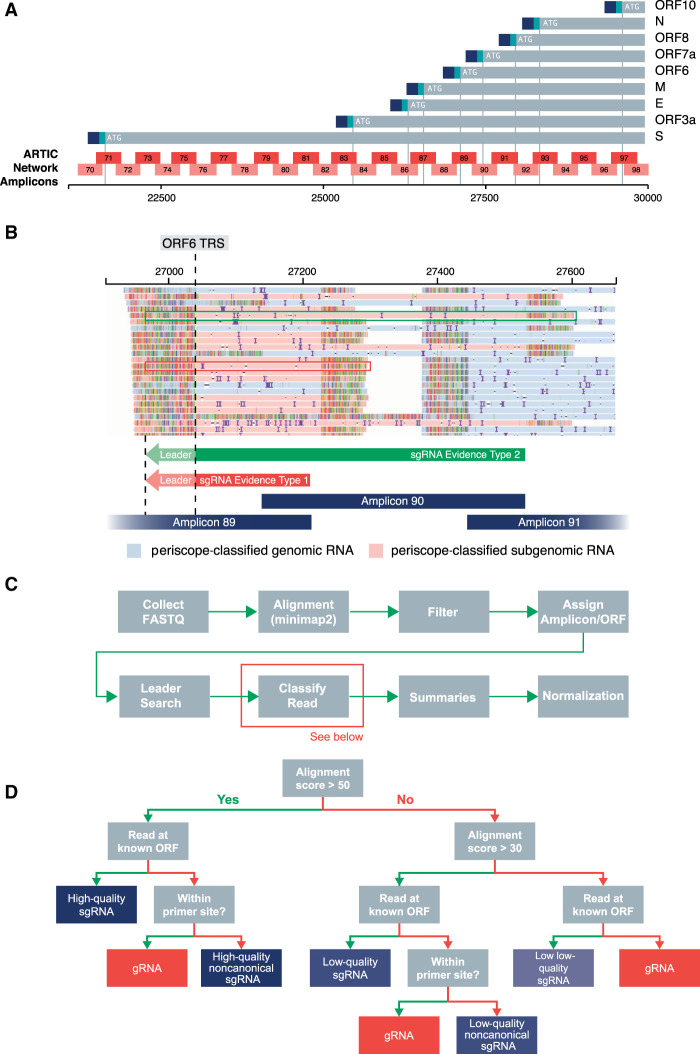
The periscope ARTIC Nanopore algorithm design details. (*A*) ARTIC network amplicon layout with respect to ORF TRS positions of SARS-CoV-2. Blue and aqua at the end of each ORF signifies leader and TRS, respectively. (*B*) Read pileup at ORF6 TRS showing two types of reads that support the existence of sgRNAs. Type 1 (red) is results from 3′→5′ amplification from the closest primer to the 3′ of the TRS site, and type 2 (green) is results from 3′→5′ amplification from the adjacent amplicons 3′ primer (i.e., the second closest 3′ primer). (*C*) Overview of the periscope workflow. (*D*) Decision tree for read classification. Green arrow denotes a “yes” for the step-in question; namely, if the read is at a known ORF start site, a green arrow is used; if not, a red arrow for “no” is used.

Previous studies of SARS-CoV-2 sgRNA have used methods that specifically detect expressed RNA, such as direct RNA sequencing of cultured cells infected with SARS-CoV-2 (Davidson et al. 2020; Kim et al. 2020; Taiaroa et al. 2020) or more traditional total poly(A) RNA-seq (Finkel et al. 2020). We hypothesized that we could detect and quantify the levels of sgRNA to both identify novel noncanonical sgRNA and provide an estimate of ORF sgRNA expression in SARS-CoV-2 sequence data. Here we present a tool for these purposes and its application to 1155 ARTIC Nanopore-generated SARS-CoV-2 sequences derived from clinical samples in Sheffield, United Kingdom, and validate our findings in data from independent SARS-CoV-2 sequences from Glasgow, United Kingdom, in addition to Illumina data generated with bait capture and metagenomic approaches.

## Results

### Evidence for sgRNA

We designed a tool, periscope, to reanalyze raw data from SARS-CoV-2 isolates to identify sgRNA based on the detection of the leader sequence at the 5′ end of reads as described previously (Leary et al. 2020).

#### ARTIC network Nanopore sequencing data

The recommended bioinformatics standard operating procedure to process ARTIC network sequencing data to produce a consensus sequence involves selecting reads between 400 and 700 bp and the trimming of the primer and adapter sequence. In most cases, this removes reads that might provide evidence for sgRNA. Mapping raw data from this protocol reveals the presence of reads at ORF TRS sites, which are sometimes shorter (Supplemental Fig. S1, sgRNA; Supplemental File S15) than the full ARTIC Network amplicon and contain leader sequence at their 5′ end. We believe these reads are the result, in the case of pool 1, priming from primer 1 of the pool, which is homologous to most of the leader sequence. We also see, in both pools, unidirectional amplification from the 3′ primer, which results in a truncated amplicon when the template is a sgRNA (Fig. 1B). We also observe longer reads that are the result of priming from the 3′ end of the adjacent amplicon (Fig. 1B).

To separate gRNA from sgRNA reads, we use the following workflow using Snakemake (Köster and Rahmann 2012); raw ARTIC Network Nanopore sequencing reads that pass QC are collected and aligned to the SARS-CoV-2 reference; and reads are filtered out if they are unmapped or supplementary alignments (reads with an alternate mapping location). We do not perform any length filtering. Each read is assigned an amplicon. We search the read for the presence of the leader sequence (5′-AACCAACTTTCGATCTCTTGTAGATCTGTTCT-3′) using a local alignment. If we find the leader with a strong match, it is likely that that read is from amplification of sgRNA. We assign reads to an ORF. By using all of this information, we then classify each read into genomic, canonical sgRNA or noncanonical sgRNA (Fig. 1D) and produce summaries for each amplicon and ORF, including normalized pseudoexpression values. sgRNA reads are binned into either high quality (HQ), where the leader alignment score is 50 or more; low quality (LQ), where the leader alignment score is 30 or more; or low, low quality (LLQ), where the read still begins at a known ORF start site (Fig. 1D).

#### Illumina sequencing data

Next, we wanted to investigate whether we could use a similar method to Illumina sequencing data. Illumina sequencing data for SARS-CoV-2 has been generated using three main approaches: amplicon based (ARTIC Network), bait capture based, or metagenomics on in vitro samples. Here we describe sgRNA detection in both Illumina bait-based capture and Illumina metagenomic data from in vitro experiments. The reads from these techniques tend to have differing amounts of leader at the 5′ end of the reads (Supplemental Fig. S2A). This is owing to library preparation methods used in these workflows. We therefore implemented a modified method for detecting sgRNA to ensure that we could capture as many reads originating from sgRNA as possible. In our Illumina implementation (Supplemental Fig. S2B,C), we extract the soft-clipped bases from the 5′ end of reads and use these in a local alignment to the leader sequence. In addition to adjusting the leader detection method, we also process mate pairs, ensuring both reads in the pair are assigned the same status.

### Detection of sgRNA

**Figure 2.**
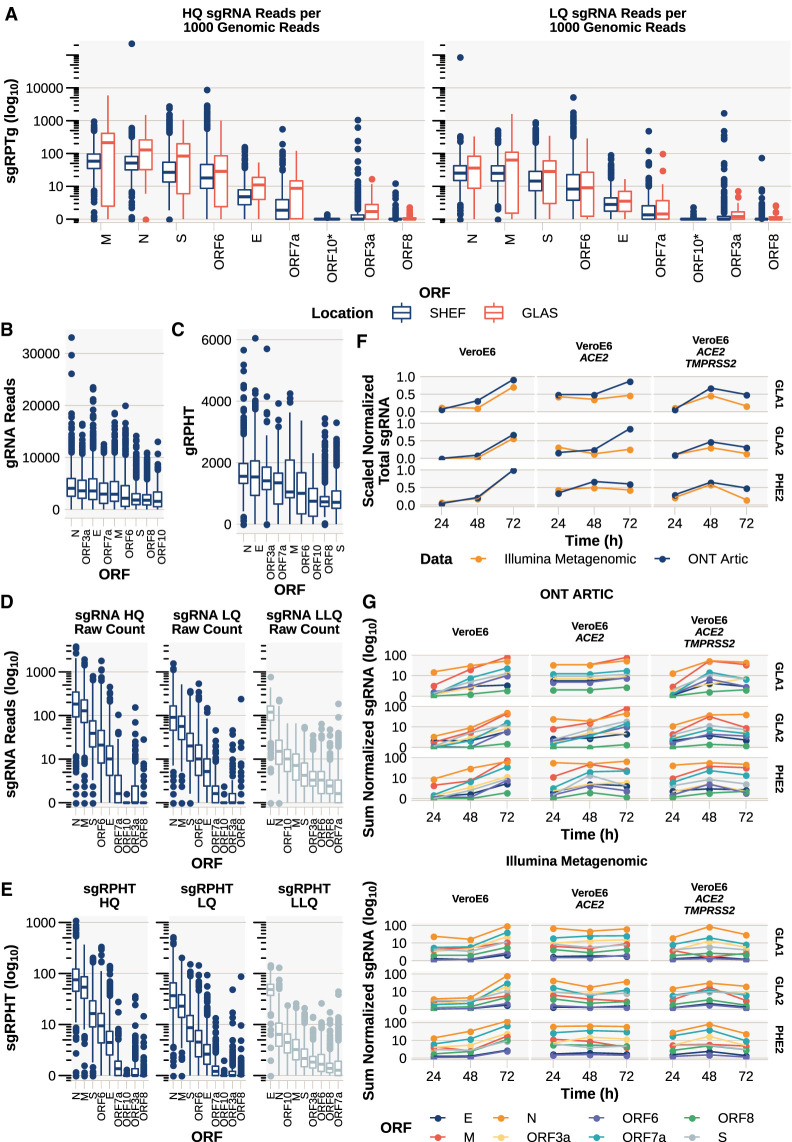
In vivo and in vitro detection and quantification of canonical sgRNA in SARS-CoV-2. (*A*) The abundance of sgRNA detected for each ORF normalized per 1000 gRNAs from Oxford Nanopore Technologies (ONT) ARTIC data from both Sheffield (*n* = 1155) (Supplemental File S1) and Glasgow (*n* = 55) (Supplemental File S7). (sgRPTg) sgRNA reads per 1000 gRNA reads. Ordered by median. See Supplemental Figure S3 for ORF10 investigation. (*B*) Number of reads supporting gRNA at each ORF. If multiple amplicons cover the ORF, then this represents the sum of reads for those amplicons. (*C*) gRNA reads normalized per 100,000 mapped reads (gRPHT) at each ORF. (*D*) Raw counts of sgRNAs. (*E*) sgRNA normalized to total mapped reads. (sgRPHT) sgRNA reads per 100,000 mapped reads. (*F*,*G*) In vitro infection time course with three SARS-CoV-2 viral isolates (GLA1, GLA2, and PHE2) in either VeroE6 cells, VeroE6 expressing *ACE2*, or VeroE6 expressing *ACE2* and *TMPRSS2*, with total RNA collected and sequenced at 24, 48, and 72 h after infection, sequenced using either ONT ARTIC (Supplemental File S6) or Illumina Metagenomic approaches (Supplemental File S5). (*F*) The sum of all normalized (to total mapped reads to allow direct comparison across ONT ARTIC and Illumina Metagenomic methods) sgRNA in each technology scaled to one. (*G*) Normalized quantity (to total mapped reads) of each canonical sgRNA in each technology. (*Top*) ONT ARTIC; (*bottom*) Illumina Metagenomic.

We were able to detect sgRNAs with a high leader alignment score from all canonical ORFs in multiple samples (Fig. 2; Supplemental Table S1; Supplemental File S1). As shown in Figure 2A, sgRNA from the N and M ORFs were the most abundant sgRNA (dependent on normalization method), with N being found in 97.3% of Sheffield samples, consistent with published reports in vitro (Alexandersen et al. 2020; Finkel et al. 2020; Kim et al. 2020). To show that the levels of sgRNA detected in the Sheffield data set were not site specific, we applied periscope to an independent data set of 55 ARTIC Network Nanopore sequenced SARS-CoV-2 samples from Glasgow, United Kingdom (Fig. 2A; Supplemental File S7).

Like previously published reports (Alexandersen et al. 2020; Finkel et al. 2020; Kim et al. 2020; Taiaroa et al. 2020), we were unable to find strong evidence of sgRNA supporting the presence of ORF10 (Fig. 2A; Supplemental Table S2) with only 0.95% of samples containing HQ or LQ sgRNA calls at this ORF. We aligned the 12 reads from these samples to a reference composing of ORF10 and leader (Supplemental Fig. S3). On manual review of these results, 10 (four HQ) of these reads are falsely classified as sgRNA. Two reads remain: one read from each of the samples SHEF-C0840 and SHEF-C58A5. These reads could represent ORF10 sgRNA as they have an almost complete match to the leader and the remainder of the reads is a strong match to ORF10.

### Normalization of subgenomic read abundance

Beyond detecting the presence of reads that are a result of amplification of sgRNA (Fig. 2D), we hypothesized that we could quantify the level of sgRNA present in a sample using either total mapped reads or gRNA reads from the same amplicon as denominators for normalization. Normalization of this kind would be analogous to traditional RNA-seq analysis in which reads per million (RPM) are calculated to allow comparisons between data sets where the number of reads affect the amount of each transcript detected. In the case of ARTIC Network Nanopore sequencing data, which involves polymerase chain reaction (PCR) of small (∼400-bp) overlapping regions of the SARS-CoV-2 genome, amplification efficiency of each amplicon should also be taken into account. Because we have a median of 258,210 mapped reads for samples from the Sheffield data set (Supplemental Fig. S4), we normalized both gRNA or sgRNA per 100,000 mapped reads (gRNA reads per 100,000 [gRPHT] or sgRNA reads per 100,000 [sgRPHT], respectively) (Fig. 2C,E).

In our second approach, because of differences in amplicon performance in the ARTIC PCR protocol that lead to coverage differences in the final sequencing data (Fig. 2B,C), we determine the amplicon from which the sgRNA has originated, using methods from the ARTIC Network Field Bioinformatics package (2020). We then normalize the sgRNA per 1000 gRNA reads from the same amplicon. If a sgRNA has resulted from more than one amplicon (Fig. 1B), the resulting normalized counts from each amplicon are summed, giving us sgRNA reads per 1000 gRNA reads (sgRPTg) for every ORF (Fig. 2A). Periscope outputs the results from both methods of normalization so that the user can decide which is more appropriate in their case and determine whether the conclusions of their analysis are consistent across both approaches.

For Illumina data, we applied one further normalization technique to allow the normalization of bait-based capture and metagenomic data. Efficiency of capture varies between probes and designs. For metagenomic data, natural fluctuations in coverage owing to sequence content can exist, therefore, to try and account for this, we took the median coverage for the region around each canonical ORF start site (±20 bp) as the denominator in the normalization of these data.

### sgRNA detection in vitro

The kinetics of sgRNA expression during the course of a SARS-CoV-2 infection is still not well understood. We applied periscope to data generated from an infection time course (Fig. 2F,G). We used both Illumina metagenomic (Supplemental File S5) and Nanopore ARTIC sequencing data (Supplemental File S6) from an in vitro model of SARS-CoV-2 infection. Wild-type (WT) VeroE6, VeroE6 expressing *ACE2*, and VeroE6 expressing both *ACE2* and *TMPRSS2* were infected with three different SARS-CoV-2 viral isolates—PHE2 (WT), GLA1 (D614G), and GLA2 (N439K and D614G) (Supplemental Table S9)—and RNA collected for sequencing at 24, 48, and 72 h. We normalized both data sets to the total mapped reads (per 100,000) to allow direct comparison. The scaled normalized total sgRNA level detected using periscope on both sequencing technologies is similar (Fig. 2F), indicating the pattern of expression is maintained between technologies. ORF N remains one of the highest expressed ORFs in both data sets (Fig. 2G). The addition of *ACE2* and a combination of *ACE2* and *TMPRSS2* results in a clear difference in the kinetics of all sgRNA over WT. These data suggest that the peak of sgRNA expression is expedited by the addition of *ACE2* and *TMPRSS2*.

### Technical replicates and batch effects

**Figure 3.**
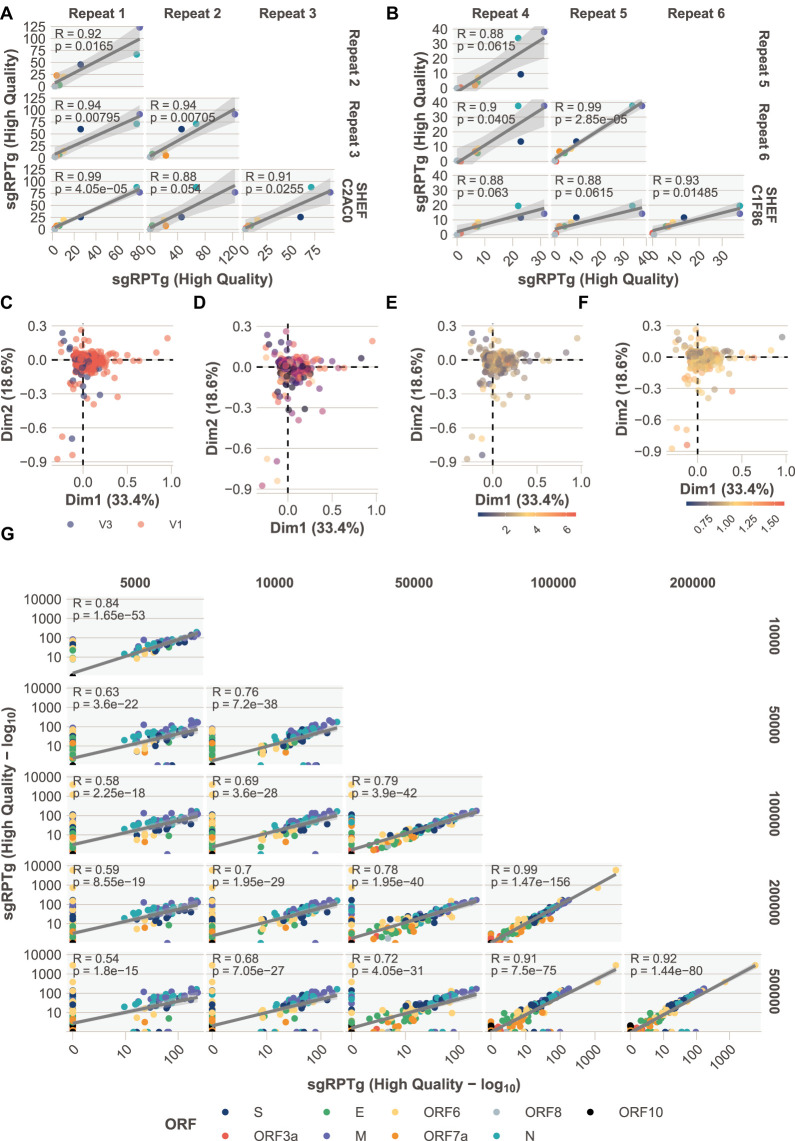
Technical replicates, detection limit, and batch effects. (*A*,*B*) Four technical replicates of two samples additional to the Sheffield cohort (Supplemental File S4). Pearson correlation coefficients between sgRPTg *P*-values adjusted with Bonferroni correction. (ORFs colored according to legend in *G*.) (*C*–*F*) Unsupervised principal component analysis (Supplemental File S8) colored by ARTIC primer version V1 or V3 (*C*), sequencing run (*D*) where the color denotes a different run, total mapped read count (scale = 100,000 reads; *E*), or normalized E gene cycle threshold (Ct) value (*F*). (*G*) Downsampling of reads from 23 high-coverage (more than 1 million mapped reads) (Supplemental File S3) samples. The number of reads provided as input to periscope was downsampled with seqtk to 5, 10, 50, 100, 200, and 500 thousand reads.

To assess the reproducibility of sgRNA analysis using ARTIC Network Nanopore sequencing data and periscope, we analyzed two samples that were subject to four technical replicates each; cDNA was independently prepared from the same swab extracted RNA, and subject to independent amplification using the recommended ARTIC Network PCR and sequenced (Fig. 3A,B; Supplemental File S4). The Pearson correlation coefficient (R) is ≥0.88 for all normalized sgRNA abundances between replicates from the same sample.

Next, we treated our sgRNA abundance values like an RNA-seq data set and asked whether other factors could be influencing expression. To do this, we used an unsupervised principal component analysis (PCA) (Fig. 3C–F; Supplemental File S8) and colored samples by the different categorical variables that could affect expression (batch effects) like sequencing run, the ARTIC primer version, the number of mapped reads, and ORF E gene cycle threshold (e.g., Ct, diagnostic test, normalized to *RPPH1* Ct value; see Methods) as this is an indicator of the amount of virus present in an isolate and a proxy for quality (Fig. 3F). There are no significant clusters between any of the above variables and the expression values in the PCA analysis.

### Lower limit of detection

The number of reads generated from any sequencing experiment is likely to vary between samples and between runs. The median mapped read count in the Sheffield data set is 258,210 but varies between 9105 and 3,260,686 (Supplemental Fig. S4). In our experience, we generally see much lower total amounts of sgRNA compared with their genomic counterparts; therefore, its detection is likely to suffer when a sample has lower amounts of reads (Supplemental Fig. S5). To determine the effect of lower coverage on the detection of sgRNA, we downsampled 23 samples that had more than 1 million mapped reads to lower read counts with seqtk (https://github.com/lh3/seqtk, accessed November 2020). We chose high (500,000 reads), medium (200,000 and 100,000), and low read counts (50,000, 10,000, and 5000) and ran periscope on this downsampled data (Supplemental File S3). In the absence of a ground truth, we performed pairwise correlation on the abundance of sgRNA between downsampled data sets (Fig. 3G; Supplemental File S3). If coverage did not affect the abundance estimates, then all coverage levels would show a high correlation coefficient (Pearson) when compared with each other (*R* > 0.7, adjusted *P* < 0.01). As expected, lower counts of the 5000 and 10,000 reads do not correlate with those generated from 100,000, 200,000, and 500,000 reads (*R*^2^ < 0.7). Samples with 50,000 reads seem to perform well compared with 100,000, 200,000, and 500,000 reads, with an *R*^2^ of 0.94, 0.89, and 0.89, respectively.

### Noncanonical sgRNA

**Figure 4.**
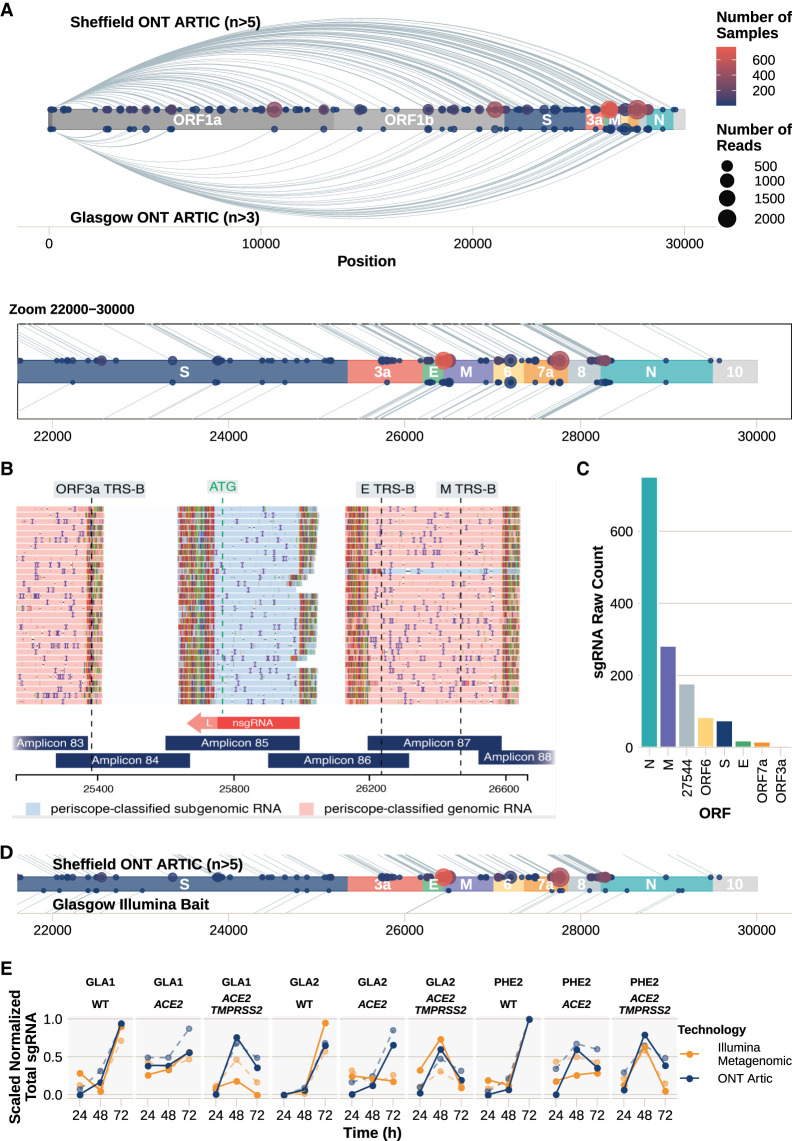
Noncanonical sgRNA. We classified reads as supporting noncanonical sgRNA as described in Figure 1D (Supplemental File S2). (*A*) Plot showing the number of samples with each noncanonical sgRNA detected in the ARTIC Nanopore data. Size of the point represents the number of reads, and the color indicates the number of samples in which noncanonical sgRNA was found. Lines connecting points represent the sgRNA product of discontinuous transcription. Those detected in Sheffield samples are *above* the genome schematic; in Glasgow, *below*. (*Inset*) Zoomed-in region between nucleotides 22,000 and 30,000. (*B*) Noncanonical sgRNA with strong support in SHEF-C0118 at position 25,744. (*C*) Raw sgRNA levels (HQ and LQ) in SHEF-C0118 show high relative amounts of this noncanonical sgRNA at position 25,744. (*D*) Zoomed-in region between nucleotides 22,000 and 30,000 of the SARS-Cov-2 genome, showing noncanonical sgRNA in the Sheffield ONT data set (*top*) compared with the noncanonical sgRNA detected in the Illumina bait capture data from Glasgow (Supplemental File S11). (*E*) Noncanonical sgRNA levels (solid lines) compared with canonical (dashed lines) in an in vitro model of SARS-CoV-2 infection measured with both Illumina metagenomic sequencing (orange) and ONT Artic (blue). Total sgRNA levels are normalized per 100,000 mapped reads and scaled within each data set for comparison.

In addition to estimating sgRNA for known ORFs, we can use periscope to detect novel, noncanonical sgRNA (Fig. 4; Supplemental File S2). We previously applied periscope to detect one such novel sgRNA (N*), which is a result of the creation of a new TRS site by a triplet variation at position 28881 to 28883, which results in production of a truncated N ORF (Leary et al. 2020). To classify sgRNA as noncanonical, supporting reads must fulfill two criteria: First, the start position does not fall in a known TRS-B region (±20 bp from the leader junction); and second, in Oxford Nanopore Technologies (ONT) data, the start position must not fall within ±5 bp from a primer sequence. We chose to implement the second criteria because we noticed a pattern of novel sgRNAs being detected at amplicon edges owing to erroneous leader matches to the primer sites.

We found evidence of noncanonical sgRNAs supported by two or more reads in 913 samples (Supplemental Fig. S6). A large quantity of these recurrent noncanonical sgRNAs cluster around known TRS-B sites (Fig. 4A), although we see enrichment of noncanonical sgRNAs at other sites throughout the genome (Fig. 4A; Supplemental Fig. S6).

In particular, SHEF-C0118 contains 177 reads (HQ and LQ) that support a noncanonical sgRNA at position 25,744 between ORFs 3a and E (Fig. 4B,C). The number of reads for this noncanonical sgRNA at 25,744 is high compared with canonical sgRNA from the same sample (Fig. 4C; Supplemental Tables S3, S4). Twenty-six samples contain one read supporting this noncanonical sgRNA, with four samples with more than one read. In another example, there are 377 samples that have evidence of one or more reads for a noncanonical sgRNA at position 10,639 (HQ or LQ; of these 155 have evidence for two or more reads ±5 bp from 10,639) (Supplemental Fig. S7A,B). In this case, there is a TRS-like sequence close to the leader in this noncanonical sgRNA; ACGAAC → ACG**G**AC. Two samples have significant support with 103 HQ reads each (SHEF-CE04A, SHEF-CA0D5 (Supplemental Tables S5, S8). It is possible that this represents an independent ORF1b sgRNA. Furthermore, there are 226 samples that have evidence for a noncanonical sgRNA at position 5785 (one or more reads; 62 with two or more reads, ±5 from 5785) (Supplemental Fig. S7C,D), where there is no core TRS sequence present and there does not appear to be a productive start codon.

#### Noncanonical sgRNAs across centers and technologies

To show the detection of noncanonical sgRNA was not a phenomenon of the Sheffield data set and ARTIC Nanopore method alone, we repeated the above analysis on both Nanopore and Illumina data from Glasgow, United Kingdom (Fig. 4A,D; Supplemental Fig. S8E; Supplemental Files S10, S11).

The noncanonical sgRNA at 5785 is found in 15/55 samples from this data set, and seven of those have multiple read support (Supplemental Table S6). This sgNA is also found with three reads in the Illumina sample CVR201. The noncanonical sgRNA found at 10,639 is found in 13/55 samples, and five of those have multiple read support (Supplemental Table S7); 10,639 is also found in Illumina sample CV196 with one read supporting; and 25,744 is supported by one HQ sgRNA read in one sample (CVR2185) from the Glasgow ONT data set.

To determine if there were any differences in noncanonical sgRNA over time, we applied the same analysis to the in vitro system described earlier (Fig. 4E; Supplemental Fig. S9; Supplemental Files S12, S13). Normalized and scaled noncanonical (solid lines) show that noncanonical sgRNA has comparable kinetics to canonical sgRNA (dashed lines), which appear to be dependent on the virus, cell line, and time since infection. This data set shows that although proportions of noncanonical sgRNA are similar (Supplemental Fig. S5), Illumina metagenomic data appear to be more sensitive (Supplemental Figs. S5, S9, S10) with a greater number of noncanonical sgRNAs detected.

### Variants in sgRNA

**Figure 5.**
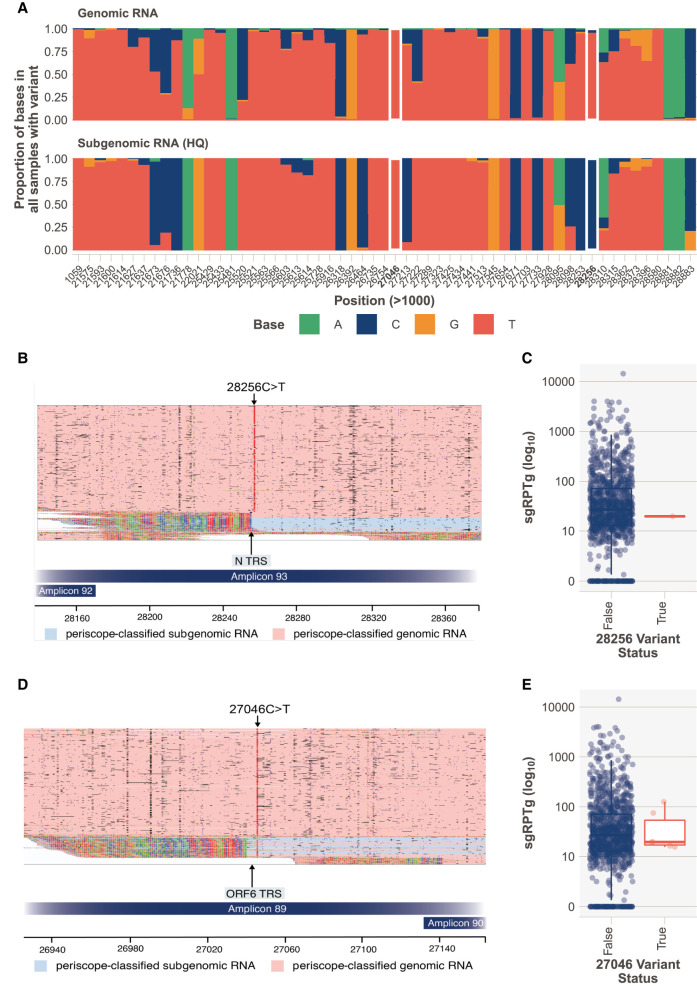
Variants in sgRNA. (*A*) Base frequencies at each of the variant positions called by ARTIC in each sample (multiple samples can be represented at one position), split by read class. White rectangles represent variants detailed in *B* and *C*. (*B*) SHEF-C0F96 has a 28,256C > T variant, of high quality that sits in the ORF N TRS sequence. This variant is not present in sgreads. (*C*) Normalized sgRNA expression (sgRPTg) for the N ORF in samples with the variant and without. N expression is one of the lowest in the cohort. (*D*) SHEF-C0C35 has 27,046C > T variant of high quality that sits in the TRS sequence. This variant is present in both gRNA and sgRNA. (*E*) ORF6 expression levels in samples with 27046C > T.

An advantage of having reads from both gRNA and sgRNA is the ability to examine how genomic variants are represented in sgRNAs. For variants found in our isolates by the ARTIC Network nanopolish (Simpson 2018) pipeline, we interrogated the bases called (pysam [Gilman et al. 2019] pileup) at the variant position in gRNA and sgRNA to determine if there were detectable differences (this tool is integrated into periscope) (Fig. 5A; Supplemental Fig. S12; Supplemental File S16). As we can only discern gRNA and sgRNA from a small subset of amplicons, the chance that a variant falls within these amplicons is low, but when the two do coincide, variants called in gRNA were supported in reads from sgRNA.

In a small subset of samples, we have identified variants in the TRS sequence of some ORFs. One sample (Fig. 5B) has a variant in the N ORF TRS (SHEF-C0F96, 28256C > T. CTAAACGAAC to **T**TAAACGAAC), which is found only in gRNA but not sgRNA reads. This sample has low expression of most ORFs (ORF N shown in Fig. 5C) and has 233,127 reads, which is around the median of the cohort. However, this mutation falls outside the core TRS and read counts for sgRNA are low so this result should be treated with caution. It is possible that this represents a sequencing error in the gRNA that is not found in the sgRNA owing to the context around that position changing as a result of the inclusion of the leader sequence.

In addition, we detected a variant at 27,046C > T (ACGAAC to ACGAA**T**) in the TRS of ORF6 in six samples (Fig. 5D); this variant is present in both the gRNA and sgRNA reads. Four of these samples have low expression of ORF6 compared to the rest of the cohort (Fig. 5E), although numbers are too low to compute statistical significance.

## Discussion

We have developed periscope, a tool that can be used on nearly all publically available SARS-CoV-2 sequence data sets worldwide to detect and quantify sgRNA. Here we applied periscope to 1155 SARS-CoV-2 sequences from Sheffield, United Kingdom, and three data sets from Glasgow; 55 ARTIC network Nanopore sequences; five bait captured Illumina sequenced samples (Supplemental File S14); and Illumina metagenomic data from an in vitro SARS-CoV-2 infection model. The development of periscope was initially motivated to aid in the detection of a novel, noncanonical, sgRNA generated (N*) from a de novo TRS site as a result of the triplet mutation 28,881G > A, 28,882G > A, and 28,883G > C found in a large number of worldwide SARS-CoV-2 isolates (Leary et al. 2020).

By searching for reads containing the SARS-CoV-2 leader sequence, incorporated into all sgRNA at their 3′ ends by the SARS-CoV-2 RNA-dependent RNA polymerase, we were able to detect sgRNA representing all annotated canonical ORFs of SARS-CoV-2. ORF10 sgRNA, however, was supported by only two reads in all 1155 samples in the Sheffield data set (Supplemental Table S1). By using an in vitro SARS-CoV-2 infection system Illumina data set, we identified a further eight reads in total supporting an ORF10 sgRNA. Seven of these reads were present at 72 h after infection, the remaining read was found at 48 h. The inability to find significant support for ORF10 mirrors previous findings (Alexandersen et al. 2020; Davidson et al. 2020; Finkel et al. 2020; Kim et al. 2020) and is perhaps expected if ORF10 is indeed nonessential (Pancer et al. 2020). The abundance of other sgRNAs is in line with previously published reports of protein levels in SARS-CoV-2, with M and N showing the highest expression levels after normalization (Bouhaddou et al. 2020; Finkel et al. 2020). In the Sheffield data set, the median proportion of total sgRNA is 1.2% (Supplemental Fig. S4), which is in broad agreement with published reports, based on ORF E, that sgRNA represented 0.4% of total viral RNA (Wölfel et al. 2020). These findings were replicated in an equivalent data set of 55 ONT ARTIC samples from Glasgow. We were able to show that sgRNA analysis using periscope is reproducible, with strong correlations between sgRNA abundance levels between technical replicates.

It has been suggested that sgRNA abundance estimates from amplicon-based sequencing data are largely a function of the quality of the RNA in the initial sample, defined in one study by average read length (Alexandersen et al. 2020). The advantage of the ARTIC Network protocol over the AmpliSeq IonTorrent protocol used by the aforementioned study is that the ARTIC Network protocol has a short, consistent amplicon length (mean is 389 and standard deviation is 11.2) (Supplemental Fig. S1, gRNA). The assay is designed inherently to deal with samples with degraded RNA. Because sgRNA reads are a product of these amplicons, we do not believe degradation plays a significant role in the determination of abundance levels in our data set. Furthermore, by using E gene Ct value as a surrogate for viral load, we find only a weak correlation with the total amount of sgRNA detected (Spearman's rank correlation, rho = 0.268) (Supplemental Fig. S13A; Supplemental File S9), and this was mainly driven by outliers. Furthermore, sequence coverage across the genome (which is affected by low viral load and poor-quality RNA) is also not correlated with sgRNA amount (Spearman's rank correlation, rho = −0.012) (Supplemental Fig. S13B; Supplemental File S17). Finally on comparison with amplification-free approaches like metagenomics, we see a similar pattern of sgRNA expression.

To show the utility of periscope for the investigation of important biological questions, we applied it to sequencing data from an in vitro infection time course using VeroE6 cells that were either WT, overexpressing *ACE2*, or overexpressing *ACE2* and *TMPRSS2* (Hoffmann et al. 2020). *ACE2* and *TMPRSS2* coexpressing cells have the most obviously altered sgRNA kinetics, with the peak level of sgRNA occurring at 48 h followed by a reduction at 72 h, which is in contrast with WT cells in which sgRNA is still accumulating after 48 h. This may indicate an expedited course of active replication allowed by greater cellular permissibility with ACE2 and TMPRSS2, followed by attenuated replication in a closed in vitro model. Of note, a greater quantity of reads from the Illumina metagenomic data are classified as sgRNA compared with ONT ARTIC data (Supplemental Fig. S4), and there are some differences between the quantities of each ORF when considered individually.

Noncanonical sgRNAs are readily detected by periscope, and we present examples in which periscope was able to detect high abundances of specific noncanonical sgRNAs in a number of isolates, which could indicate some functional significance. It has previously been observed that noncanonical sgRNAs are not formed owing to a TRS-like homology (Nomburg et al. 2020). The noncanonical sgRNA at position 25,744 in SHEF-C0118 has a high relative abundance compared with the canonical sgRNAs in the same sample. There does not appear to be a canonical TRS sequence in close proximity to the leader junction, but there exists a motif that has two mismatches to the canonical TRS, A**A**GAA**T**. An ATG downstream from the leader in these reads would result in an N-terminal truncated 3a protein. Two forms of 3a protein in SARS-CoV have been noted in the literature (Huang et al. 2006). Alternatively, SARS-CoV contains a nested ORF within the 3a sgRNA, 3b (Supplemental Fig. S11), but the homolog of this protein is truncated early in SARS-CoV-2; however, others note that a protein from this truncated 3b, of only 22 amino acids in length, could have an immune regulatory function (Konno et al. 2020). This noncanonical sgRNA could indicate production of this novel 3b protein in SARS-CoV-2 independent from the 3a sgRNA, a phenomenon that has been shown to occur in SARS-CoV (Hussain et al. 2005). We cannot explain why, in this sample, this noncanonical sgRNA is present in such high abundance. There are no genomic variants that contribute to a TRS sequence, for example. We also find evidence of highly recurrent noncanonical sgRNAs that have weaker evidence like those at 10,639, which could represent an independent sgRNA for ORF 1b. Others, like those at 5785 have no apparent related ORF.

Some studies have detected the presence of an sgRNA for ORF7b (Finkel et al. 2020; Kim et al. 2020); we also explored this possibility (Supplemental Fig. S11C–E). We are able to detect noncanonical sgRNAs just 3′ of the predicted start codon of ORF7b (27,760 and 27,761) and at least 10 bases downstream from a predicted TRS-B site (Yang et al. 2020). These sgRNAs have strong support in both the Sheffield (Supplemental Fig. S11D), 668/1155 samples (one or more HQ sgRNA, median of two reads per sample, and a maximum of 133), and Glasgow data sets, 44/55 samples (one or more HQ sgRNA, median of 1.5 reads per sample, and a maximum of 12). Raw reads from SHEF-BFF12 show that the leader body junction in these sgRNAs does indeed exclude the start codon and in fact includes four additional bases not present in the genome (Supplemental Fig. S11E). It is possible that this sgRNA encodes a protein from another ATG site downstream from the leader–body junction.

We were able to detect a number of these recurrent noncanonical sgRNAs in the data from Glasgow, showing that these noncanonical sgRNAs are unlikely to be sequencing artifacts and may represent favored sites for noncanonical sgRNA generation during SARS-CoV-2 replication for as yet unexplained reasons. We speculate that the diversity of noncanonical sgRNA seen in our data set, which is most comparable to total RNA-seq (Wyler et al. 2021) than direct RNA-seq, is a function of (1) the number of samples analyzed and (2) the varied and unknown length of the infection at the time of sampling. These findings illustrate that although much is not known about the expression of noncanonical sgRNA, periscope could help define and quantify these noncanonical transcripts in order to explore their relevance in SARS-CoV-2 pathogenesis.

The COVID-19 Genomics UK (COG-UK) Consortium (2020) in the UK, alone, has 16,826 ARTIC Nanopore and 69,969 Illumina sequences (correct October 29, 2020), whereas internationally GISAID contains thousands more similar data sets (8775 with “Nanopore” in the metadata and 3660 list “ARTIC” as of June 14, 2020). The application of periscope could therefore provide significant insights into the sgRNA architecture of SARS-CoV-2 at an unprecedented scale. Furthermore, periscope can be provided new primer/amplicon locations for PCR-based genomic analysis protocols, and we have shown that it can be applied to metagenomic sequencing methods without prior species-specific genome amplification. Periscope could also be applied to sequencing data from other viruses where discontinuous transcription is the method of gene expression.

Periscope offers an opportunity to further understand the regulation of the SARS-CoV-2 genome by identifying and quantifying sgRNA. Applying it to the vast amount of SARS-CoV-2 sequencing data sets that have been generated worldwide during this unprecedented public health crisis could uncover critical insights into the role of sgRNA in SARS-CoV-2 pathogenesis.

## Methods

### Sheffield SARS-CoV-2 sample collection and processing

One thousand one hundred fifty-five samples from 1155 SARS-CoV-2-positive individuals were obtained from either throat or combined nose/throat swabs. Nucleic acids were extracted from 200 µL of each sample using MagNA Pure extraction platform (Roche Diagnostics). SARS-CoV-2 RNA was detected using primers and probes targeting the E gene and the RdRp genes of SARS-CoV-2 and the human gene *RPPH1* to allow normalization, for routine clinical diagnostic purposes, with thermocycling and fluorescence detection on ABI Thermal Cycler (Applied Biosystems) using previously described primer and probe sets (Corman et al. 2020).

### Sheffield SARS-CoV-2 isolate amplification and sequencing

Nucleic acids from positive cases underwent long-read whole-genome sequencing (ONT) using the ARTIC Network protocol (accessed April 19, 2020, https://artic.network/ncov-2019, https://www.protocols.io/view/ncov-2019-sequencing-protocol-v3-locost-bh42j8ye). In most cases, 23 isolates and one negative control were barcoded per 9.4.1D Nanopore flow cell. Following base-calling, data were demultiplexed using ONT Guppy (‐‐require-both-ends). Reads were filtered based on quality and length (400–700 bp) and then mapped to the Wuhan reference genome (MN908947.3) and primer sites trimmed. Reads were then downsampled to 200× coverage in each direction. Variants were called using nanopolish (Simpson 2018).

### In vitro SARS-CoV-2 infection model

Two derivatives and an unaltered lineage of the VeroE6 African green monkey kidney cell line were used in this study. One VeroE6 cell line was manipulated to overexpress the angiotensin-converting enzyme 2 (ACE2) receptor. In addition to ACE2 overexpression, a third line was also induced to overexpress the transmembrane serine protease 2 (TMPRSS2). These cell lines are referred to as VeroE6, VeroE6 *ACE2*, and VeroE6 *ACE2 TMPRSS2*, respectively. Methods for the production and the validation of these cell lines are fully described by Rihn et al. (2021). SARS-CoV-2 infection of these three cell lines were set up with three viral isolates (PHE2, GLA1, and GLA2) (Supplemental Table S9) and supernatant harvested at 24, 48, and 72 h for viral RNA extraction and sequencing.

### Illumina metagenomic sequencing

This protocol was applied to virus isolates propagated in vitro. Extracted nucleic acid was incubated with DNase I (Thermo Fisher Scientific AM2222) for 5 min at 37°C. After DNase treatment, the samples were purified using Agencourt RNA clean AMPure XP beads (Beckman Coulter A63987), following the manufacturer's guidelines, and quantified using the Qubit dsDNA HS Kit (Thermo Fisher Scientific Q32854). cDNA was synthesized using SuperScript III (Thermo Fisher Scientific 18080044) and a NEBNext Ultra II non-directional RNA second strand synthesis module (New England Biolabs E6111L), as per the manufacturer's guidelines.

Samples were further processed using the Kapa LTP library preparation kit for Illumina Platforms (Kapa Biosystems KK8232). Briefly, the cDNA was end-repaired and the protocol followed through to adapter ligation. At this stage, the samples were uniquely indexed using the NEBNext multiplex oligos for Illumina 96 unique dual index primer pairs (New England Biolabs E6442S), with 15 cycles of PCR performed.

All amplified libraries were quantified by a Qubit dsDNA HS Kit and run on the Agilent 4200 Tapestation system (Agilent G2991AA) using the high-sensitivity D5000 Screentape (Agilent 5067-5592) and high-sensitivity D5000 reagents (Agilent 5067-5593). Libraries were sequenced on an Illumina NextSeq 550 (Illumina SY-415-1002).

### sgRNA detection

Periscope consists of a Python-based Snakemake (Köster and Rahmann 2012) workflow, which runs a Python package that processes and classifies reads based on their configuration (Fig. 1C).

#### Preprocessing

##### Nanopore

Pass reads for single isolates are concatenated and aligned to MN908947.3 with minimap2 (v2.17) (-ax map-ont -k 15) (Li 2018). It should be noted that adapters or primers are not trimmed. BAM files are sorted and indexed with SAMtools (Li et al. 2009).

##### Illumina

Paired-end reads, ideally before trimming, are aligned to MN908974.3 with BWA-MEM (v0.7.17) (Li and Durbin 2009) with the “Y” flag set to use soft clipping for supplementary alignments. BAM files are sorted and indexed with SAMtools.

#### Periscope: leader identification and read classification

##### Nanopore

Reads from the minimap2 aligned BAM file are then processed with pysam (Gilman et al. 2019). If a read is unmapped or represents a supplementary alignment, then it is discarded. Each read is then assigned an amplicon using the “find_primer” method of the ARTIC field bioinformatics package. We search for the leader sequence (5′-AACCAACTTTCGATCTCTTGTAGATCTGTTCT-3′) with Biopython (Cock et al. 2009) local pairwise alignment (localms) with the following settings: match +2, mismatch −2, gap -10, and extension -0.1, with score_only set to true to speed up computation. The read is then assigned an ORF using a pybedtools (Dale et al. 2011) and a BED file consisting of all known ORFs ±10 of the predicted leader/genome transition.

We classify reads as a HQ sgRNA (Fig. 1D) if the alignment score is greater than 50 and the read is at a known ORF. If the read starts at a primer site, then it is classified as gRNA; if not, then it is classified as a HQ noncanonical sgRNA supporting read. If the alignment score is greater than 30 but 50 or less and if the read is at a known ORF, then it is classified as a LQ sgRNA. If the read is within a primer site, it is labeled as a gRNA; if not, then it is a LQ sgRNA. Finally, any reads with a score of 30 or less that are at a known ORF are then classified as a LLQ sgRNA; otherwise, they are labeled as gRNA. The following tags are added to the reads for manual review of the periscope calls: XS, alignment score; XA, amplicon; AC, read class; and XO, the read ORF. Reads are binned into qualitative categories (HQ, LQ, LLQ, etc.) because we noticed that some sgRNAs were not classified as such owing to a lower match to the leader. After manual review, they are deemed bona fide sgRNA. This quality rating negates the need to alter alignment score cutoffs continually to find the best balance between sensitivity and specificity. Restricting to HQ data means that sensitivity is reduced but specificity is increased, including LQ calls will decrease specificity but increase sensitivity.

##### Illumina

Reads from the BWA-MEM-aligned BAM file are processed with pysam. If a read is unmapped or represents a supplementary alignment, then it is discarded.

The presence of soft clipping at the 5′ end of the reads is an indicator that the read could contain the leader sequence so we extract all of the soft clipped bases from the 5′ end, additionally including three further bases to account for homology between leader and genome at the N ORF (these bases would therefore not be soft clipped at this ORF). If there are fewer than six extracted bases in total, we do not process that read further as this is not enough to determine a robust match to the leader sequence. With a match score of two and a mismatch score of −2 (gap opening penalty, −20l extension, −0.1), soft clipped bases are aligned to the leader sequence with localms. Soft clipped bases that include the full ≥33 bp of the leader would give an alignment score of 66. Allowing for two mismatches, this gives a “perfect” score of 60. If the number of soft clipped bases is less, then we adjust the “perfect” score in the following way:

Perfect Score=(Number of bases soft clipped×2)−2.

This allows for one mismatch. The position of the alignment is then checked; for these bases to be classed as the leader, the alignment of the soft clipped bases must be at the 3′ end. If this is true and (Perfect Score − Alignment Score) ≤ 0, then the read is classified as sgRNA.

#### Periscope: sgRNA normalization

##### Amplicon data

Once reads have been classified, the counts are summarized and normalized. Two normalization schemes are used.

1. Normalization to total mapped reads
  Total mapped reads per sample are calculated using pysam idxstats and used to normalize genomic, sgRNA, and noncanonical sgRNA reads. Reads per hundred thousand total mapped reads are calculated per quality group:
  $$\mathrm{sgRPHT}\;\;=\;\frac{\;\;\mathrm{sgRNA}\;\;\mathrm{Read}\;\;\mathrm{Count}\;\times \;100000}{(\mathrm{Total}\;\;\mathrm{Mapped}\;\;\mathrm{Reads}\;100000}.$$
2. Normalization to genomic reads from the corresponding amplicon
  Counts of sgRNA, noncanonical sgRNA, and gRNA are recorded on a per amplicon basis, and normalization occurs within the same amplicon per 1000 gRNA reads. If multiple amplicons contribute to the count of sgRNA or noncanonical sgRNA, then the normalized values are summed:
  $$\mathrm{sgRPTG}\;=\;\frac{\mathrm{sgRNA}\;\;\mathrm{Read}\;\;\mathrm{Count}\;\;\times \;1000}{\mathrm{Total}\;\;\mathrm{Genomic}\;\mathrm{RNA}}.$$

Periscope outputs several useful files that are described in more detail in the Supplemental Material; briefly, these are periscope's processed BAM file with associated tags, a per amplicons counts file, and a summarized counts file for both canonical and noncanonical ORFs.

##### Bait-based capture and metagenomic data

Again, two schemes are used, as above, to the total amount of mapped reads and, additionally, the following is used.

3. Normalization to local coverage

For both canonical and noncanonical sgRNA normalization, we calculate the median coverage around either (1) for canonical ORFs, the TRS site ±20 bp or (2) the leader/genome junction ±20 bp and normalize the total sgRNA per 1000 reads of coverage.

$$\mathrm{sgRPTL}\;\;=\;\frac{\mathrm{sgRNA}\;\;\mathrm{Read}\;\;\mathrm{Count}\;\times \;1000}{\mathrm{Local}\;\;\mathrm{Coverage}}.$$

### Subgenomic variant analysis

A Python script is provided “variant_expression.py” that takes the periscope BAM file and a VCF file of variants (usually from the ARTIC analysis pipeline). For each position in the VCF (pyvcf) (https://github.com/jamescasbon/PyVCF, accessed November 2020) file, it extracts the counts of each base in each class of read (i.e., genomic, sgRNA and noncanonical sgRNA) and outputs these counts as a table. This tool also provides a useful plot (Supplemental Fig. S5) of the base counts at each position for each class.

### Analysis and figure generation

Further analysis was completed in R 3.5.2 (R Core Team 2020) using Rstudio 1.1.442 (Racine 2012). In general, data were processed using dplyr (v0.8.3) and figures were generated using ggplot2 (v3.3.1), both part of the tidyverse (Wickham et al. 2019) family of packages (v1.2.1). Plots were themed with the ftplottools package (v0.1.5). GGally (v2.0.0) ggpairs was used for the matrix plots for downsampling and repeats. When multiple hypothesis tests were performed, multiple testing correction was performed using Bonferroni. Reads were visualized in Integrative Genomics Viewer (IGV) (Robinson et al. 2011) and annotated with Adobe Illustrator. Code to reproduce the analyses and for figure generation is available as Supplemental File S18 and at GitHub (https://github.com/sheffield-bioinformatics-core/periscope-publication).

#### Principal component analysis

PCA was performed to determine if any of the experimental variables were responsible for the differences in expression values between samples. Reads from ORF1a and ORF10 amplicons were removed from the analysis, and expression values were normalized within each ORF,

$$\frac{x-\min(x)}{\max(x)-\min(x)},$$

and then row means subtracted. The “PCA” function of the R package FactoMineR (v1.41) (Lê et al. 2008) was then used to perform the PCA without further scaling and all other settings as default. The resulting PCA was plotted using the “fviz_pca_ind” function. Plots were colored according to the variable in question.

### Data cleaning and upload to ENA

Data was uploaded to European Nucleotide Archive (ENA; https://www.ebi.ac.uk/ena/browser/home) after removing human reads with dehumanizer (https://github.com/SamStudio8/dehumanizer) and then mapping the resulting reads to hg38 with BWA-MEM for Illumina data or minimap2 for ONT data. Only those reads that did not map were retained. Reads were converted from BAM to FASTQ with SAMtools bam2fq. A convenient table which converts ENA accession numbers to sample ID can be found in Supplemental File S19.

### Periscope requirements

Periscope is a wrapper for a Snakemake (Köster and Rahmann 2012) workflow with a package written in Python to implement read filtering and classification and is provided with a conda environment definition. It has been tested on a Dell XPS, core i9, 32 GB ram, 1 TB SSD running ubuntu 18.04, and was able to process approximately 100,000 reads per minute. Periscope installation requires conda (e.g., Miniconda, https://docs.conda.io/en/latest/miniconda.html, version 3.7 or 3.8). To run periscope, you will need the path to your raw FASTQ files from your ARTIC Network Nanopore sequencing run or Illumina paired-end reads (unfiltered), as well as other variables defined in the Supplemental Material and on the GitHub README.

### Ethics approval and consent

Individuals presenting with active COVID-19 disease were sampled for SARS-CoV-2 sequencing at Sheffield Teaching Hospitals NHS Foundation Trust, United Kingdom, using samples collected for routine clinical diagnostic use. This work was performed under approval by the Public Health England Research Ethics and Governance Group for the COVID-19 Genomics UK consortium (R&D NR0195).

## Data access

All raw sequencing data generated in this study have been submitted to the European Nucleotide Archive (ENA; https://www.ebi.ac.uk/ena/browser/search) under accession number PRJEB40972. The periscope source code is available as Supplemental File S20 and at GitHub (https://github.com/sheffield-bioinformatics-core/periscope).

## Competing interest statement

The authors declare no competing interests.

## Supplementary Material

Supplemental Material
