# Supplementary material for: Subgenomic RNA identification in SARS-CoV-2 genomic sequencing data
Source: Genome Res. 2021 Apr;31(4):645–58. doi: 10.1101/gr.268110.120 (PMC8015849; doi:10.1101/gr.268110.120)
Supplement: Supplemental Material [file supp_31_4_645__index.html]

Subgenomic RNA identification in SARS-CoV-2 genomic sequencing data — Supplemental Material 

# Subgenomic RNA identification in SARS-CoV-2 genomic sequencing data

## Supplemental Material

- Supplemental\_File.zip
- Supplemental\_Material\_.pdf
- Supplemental\_File\_S18.txt
